# Purification and Mechanism of Microcystinase MlrC for Catalyzing Linearized Cyanobacterial Hepatotoxins Using *Sphingopyxis* sp. USTB-05

**DOI:** 10.3390/toxins14090602

**Published:** 2022-08-31

**Authors:** Qianwen Zou, Junhui Teng, Kunyan Wang, Yiming Huang, Qingbei Hu, Sisi Chen, Qianqian Xu, Haiyang Zhang, Duyuan Fang, Hai Yan

**Affiliations:** 1 School of Chemistry and Biological Engineering, University of Science and Technology Beijing, Beijing 100083, China; 2 Beijing Royal School, Beijing 102209, China

**Keywords:** cyanobacterial hepatotoxins, microcystinase (MlrC), active site, mechanism

## Abstract

Cyanobacterial hepatotoxins, including microcystins (MCs) and nodularins (NODs), are widely produced, distributed and extremely hazardous to human beings and the environment. However, the catalytic mechanism of microcystinase for biodegrading cyanobacterial hepatotoxins is not completely understood yet. The first microcystinase (MlrA) catalyzes the ring opening of cyclic hepatotoxins, while being further hydrolyzed by the third microcystinase (MlrC). Based on the homology modeling, we postulated that MlrC of *Sphingopyxis* sp. USTB-05 was a Zn^2+^-dependent metalloprotease including five active sites: Glu56, His150, Asp184, His186 and His208. Here, the active recombinant MlrC and five site-directed mutants were successfully obtained with heterologous expression and then purified for investigating the activity. The results indicated that the purified recombinant MlrC had high activity to catalyze linearized hepatotoxins. Combined with the biodegradation of linearized NOD by MlrC and its mutants, a complete enzymatic mechanism for linearized hepatotoxin biodegradation by MlrC was revealed.

## 1. Introduction

Due to water eutrophication and global warming, cyanobacterial blooms frequently occur in various aquatic ecosystems worldwide, causing serious environmental hazards and public health threats [1,2,3]. Cyanobacteria produce and release various kinds of toxins, especially microcystins (MCs) and nodularins (NODs), into water bodies [4,5,6]. MCs and NODs have similar structures to a particular amino acid [7]. MC-LR is the most toxic of the microcystin isomers [8,9]. Studies about the biodegradation mechanism of MC-LR can be found [10,11,12], but less information is available on NODs. A NOD with acute toxicity is released by *Nodularia spumigena* [13] and its cyclic structure is stable and resistant to hydrolysis [14]. These toxins accumulate in aquatic animals and endanger human health through the food web [15]. It is worth noting that human health risks may continue to increase in the future [16,17]. Consequently, it is important to find how to decrease the toxicity of cyanotoxins effectively.

Biodegradation is a promising solution for the removal of cyanobacterial toxins, which is also not harmful to the natural environment or humans [11,18,19]. Despite this, cyanobacterial hepatotoxins can be degraded by a number of microorganisms [18,20,21,22]. Microcystinase plays a prominent role in biodegrading cyclic hepatotoxins. Not all strains of microcystinase can biodegrade MCs and NODs [23]. Four genes are involved in the degradation pathway—*mlrA, mlrB, mlrC* and *mlrD*—which form an *mlr* gene cluster and encode three hydrolysis enzymes (MlrA, MlrB and MlrC) and one oligopeptide transporter-like protein (MlrD), respectively [24]. MlrA catalyzes the first biodegradation step of hepatotoxin by cleaving the Adda–Arg peptide bond, producing a linear hepatotoxin. The linear structure needs to be hydrolyzed by MlrB and MlrC. MlrB hydrolyzes the linearized hepatotoxin, yielding a tetrapeptide, which can also be hydrolyzed by MlrC. However, Bourne et al. verified that the tetrapeptide product was detected as more toxic than the linearized hepatotoxin [25]. These enable recombinant MlrC proteins to be very helpful in the investigation of the enzyme activity and degradation mechanism.

*Sphingopyxis* sp. USTB-05 (GenBank accession number of the 16S rDNA sequences: EF607053) was isolated and identified for efficiently biodegrading cyanotoxins of both MCs and NODs [26,27]. Our previous study indicated that the MlrA of *Sphingopyxis* sp. USTB-05 was likely a glutamate protease belonging to type II CAAX prenyl endopeptidases [28]. However, research on the characterization of MlrC is hardly reported on.

This work aimed to investigate the mechanism of linear hepatotoxin biodegradation by MlrC of *Sphingopyxis* sp. USTB-05. MlrC with high activity was obtained and purified using a recombinant *E. coli* overexpressing strain. Based on the homology modeling, we postulated that MlrC was likely a Zn^2+^-dependent metalloprotease with five crucial sites: Glu56, His150, Asp184, His186 and His208.

Furthermore, site-directed mutation experiments were performed for further verification. A complete enzymatic mechanism for linear hepatotoxin biodegradation by MlrC was proposed: Asp184, His186 and His208 coordinate with Zn^2+^; Glu56 and Zn^2+^ coactivate one water molecule, then nucleophilic attack the carbonyl oxygen atom and cleave the Adda–Glu peptide bond; His150 plays a role in stabilizing the transition states. A complete picture for the structural basis of MlrC activity was exhibited and a better understanding for the enzymatic mechanism of biodegradation for hepatotoxin was provided.

## 2. Results

### 2.1. Homology Modeling of USTB-05-C

**Figure 1:**
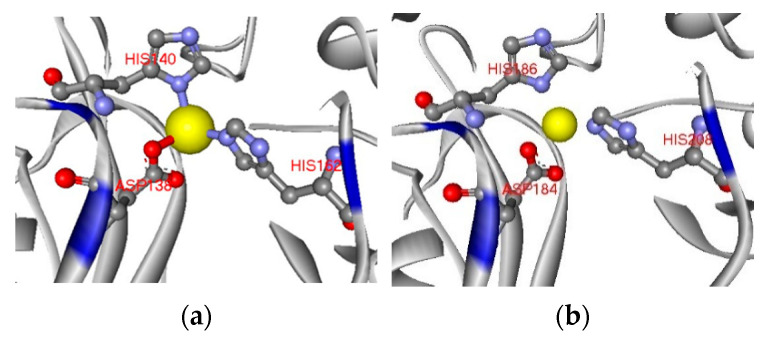
(**a**) 3IUU binds to Zn^2+^ (yellow); (**b**) USTB-05-C binds to Zn^2+^ (yellow).

The 3D structure of USTB-05-C (MlrC) was acquired with homology modeling with the Phyre^2^ engine [29]. Homology searching indicated that a putative metallopeptidase (Protein Data Bank database ID: 3IUU) had a sequence identity of 26% with MlrC. Moreover, the coverage was 91% by aligning the sequence of 3IUU and MlrC. It was implied that both proteins maintained a high conservatism of their 3D structure. The 472 residues (89% of MlrC) could be modeled with a confidence of 100%. The 3D structure of the template protease binding to Zn^2+^ is given in Figure 1a. The amino acids Asp184, His186 and His208 coordinated with Zn^2+^ in the center of 3IUU. Likewise, amino acids Asp184, His186 and His208 coordinated with Zn^2+^ in USTB-05-C (Figure 1b).

Moreover, the mechanism of typical metalloproteinase ZMPSTE24 (Protein Data Bank database ID) was referred to speculate the characteristic of USTB-05-C. For ZMPSTE24 [30], amino acids His335, His339 and Glu415 coordinated with Zn^2+^, Glu336 attacked the catalytic substrate residue and the water molecule was aligned and activated. If this residue was mutated to Ala, the activity of ZMPSTE24 would reduce to a fifth of Glu. Likewise, Asp265 and His459 are deemed to stabilize the transition state during the catalytic process [31].

### 2.2. Purification of Recombinant USTB-05-C

**Figure 2:**
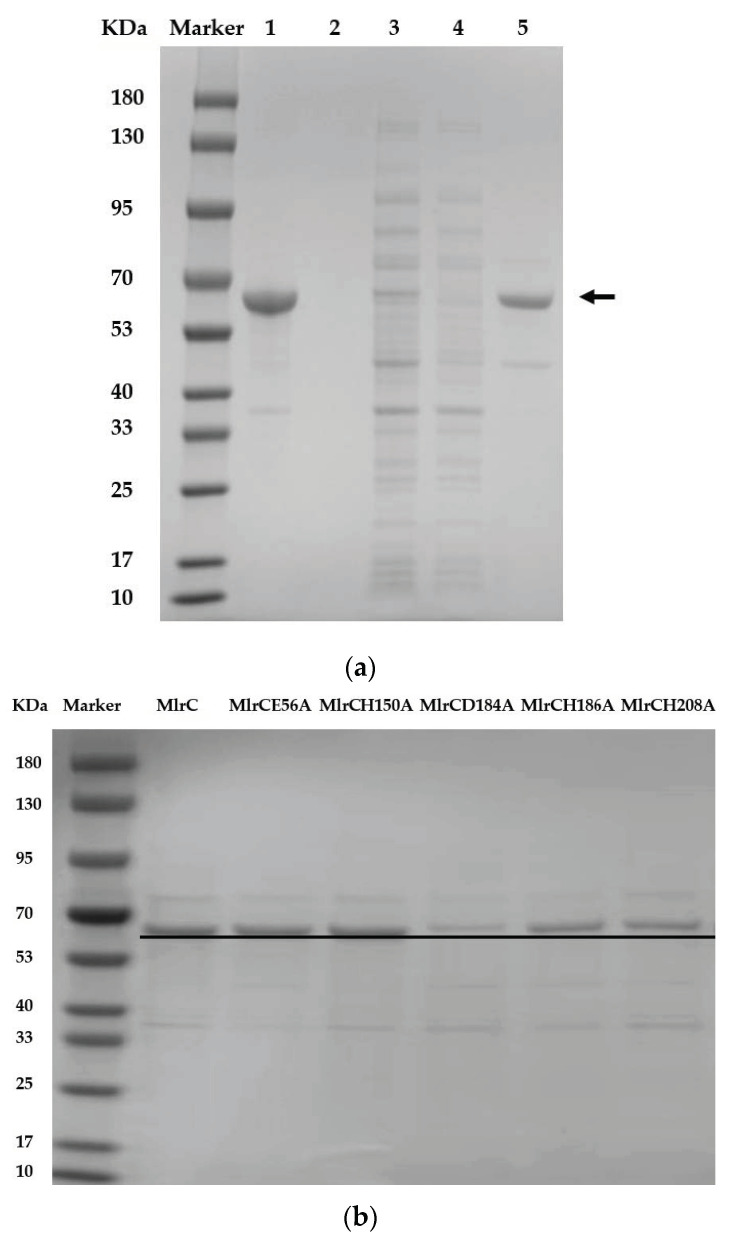
Purification of recombinant MlrC (**a**) and its site-directed mutants (**b**).

Figure 2a shows the profiles of different fractions in the steps of purification. An inclusion body in the precipitation was a major pattern of the recombinant MlrC (lane one), but a supernatant with a soluble MlrC was used (lane three) for further purification. Ultrafiltration was used to concentrate MlrC and remove the imidazole. The purified MlrC was indicated with a black arrow in lane five. By applying the washing buffer with 20 mM of imidazole, all impurities and other bound proteins were verified to be removed without MlrC elution loss (lane two), suggesting the suitability of such a washing buffer. The BCA quantification of the protein result showed that the active MlrC expression reached the concentration of 215 mg L^−1^ under optimum induction conditions. The activity of MlrC may have been weakened during purification [32], thus, it could be more advantageous and substantial to use a MlrC supernatant for functional research and practical applications than a purified enzyme.

The method of purification as above was applied to recombinant MlrC mutants. SDS-PAGE analysis verified the successful expression of six USTB-05-C mutants and a positive control (USTB-05-C) under optimum induction conditions (Figure 2b).

### 2.3. Enzyme Activity

**Figure 3:**
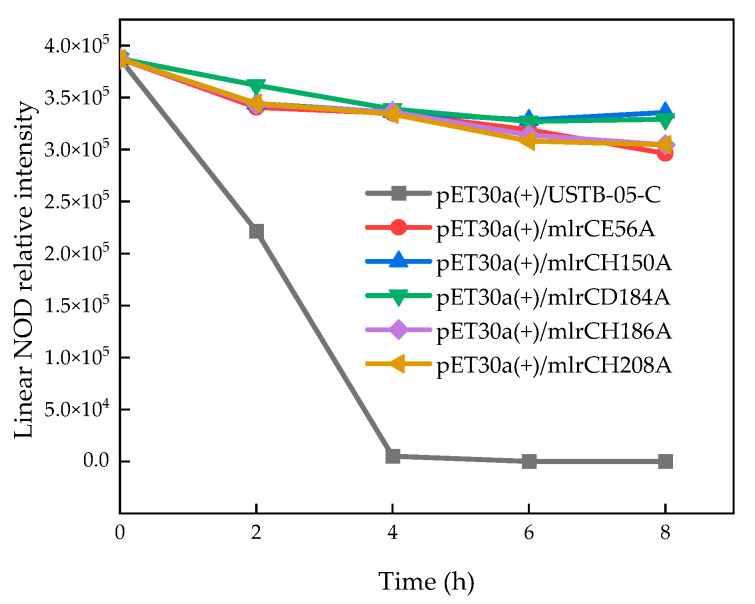
Linearized NOD biodegradation kinetics using purified enzyme from the supernatant of *E. coli* BL21 (DE3) cells transformed with *mlrC* and *mlrC* mutants.

A linear NOD was produced by catalyzing a NOD using MlrA, which was further biodegraded by MlrC (Figure 3) [33]. Purified USTB-05-C and six recombinants were diluted to the same concentration. The initial linear NOD was degraded approximately 98.7% within 4 h and completely removed within 6 h by purified MlrC. However, the activities of the mutants, including E56A, H150A, D184A, H186A and H208A, against the linear NOD were almost completely abolished, which confirmed that these five sites were crucial for the USTB-05-C to biodegrade the linear NOD, in good agreement with the work on MlrC from *Novosphingobium* sp. THN1 by Wang et al. [12].

## 3. Discussion

At present, the main methods of the removal of cyanobacterial hepatotoxins include using a physical pathway, chemical pathway and biodegradation [34,35,36,37]. Biodegradation is a promising solution to remove cyanobacterial hepatotoxins produced by algal blooms. The promising strain *Sphingopyxis* sp. USTB-05 has a strong ability to biodegrade cyanobacterial toxins, and has at least three enzymes encoded by biodegradation genes [38,39]. The first microcystinase (MlrA) is crucial for catalyzing the ring opening of cyclic hepatotoxins by cleaving the Adda–Arg peptide bond, and then, the linearized MCs are biodegraded by the enzyme MlrB, giving rise to a tetrapeptide. The enzyme MlrC can hydrolyze both the tetrapeptide and linearized product at the Adda–Glu bond to isolate Adda. Similar results were explained in a number of strains [26,32,38,39,40]. Isolated Adda has been proven to be virtually harmless [41]. Hence, MlrA and MlrC are the most important in the process of detoxification for MCs and NODs. Studies about the properties and active sites of MlrA have been reported previously [10,28], but less information exists concerning the characterization of MlrC for catalyzing linearized cyanobacterial hepatotoxins, especially NODs. Based on the above, the catalytic function of USTB-05-C towards linearized NODs deserves urgent study.

The structure of a protein is the basis of its function; here, the phyre^2^ online tool was used to analyze the structural properties of USTB-05-C. The structure of MlrC is highly homologous with a chain of 3IUU. Based on 3IUU as a template, the 3D structure of MlrC was predicted. Since the template is a class of metalloproteinases, it was predicted that MlrC also possessed the properties of metalloproteinase. It involved activating the water molecule and catalyzing the Adda–Glu peptide between the linear cyclopeptide toxins by binding with Zn^2+^.

**Figure 4:**
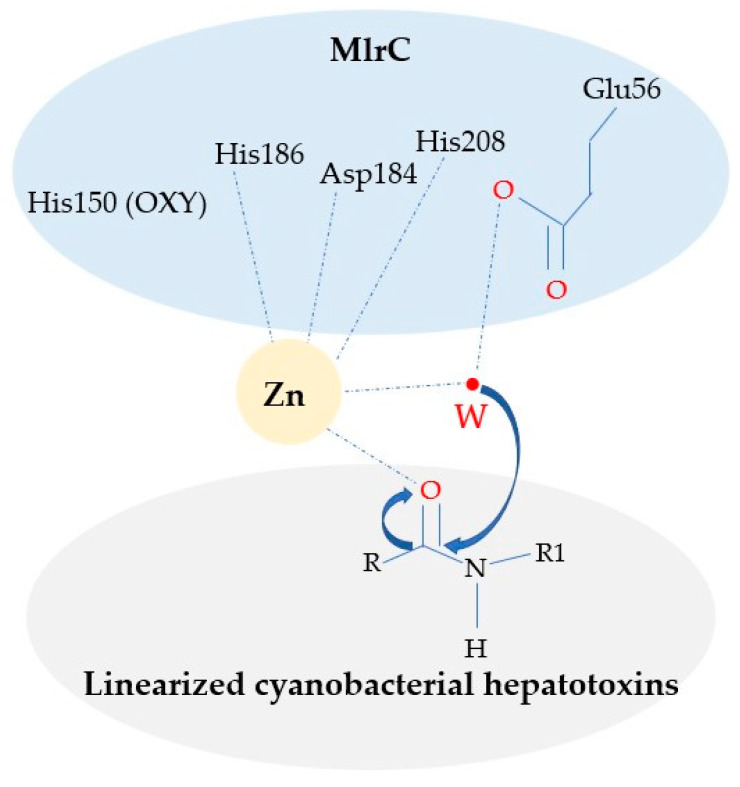
Mechanism of MlrC for biodegrading linearized cyanobacterial hepatotoxins.

The catalytic sites and characteristics of ZMPSTE24 were understood; hence, the enzymatic mechanism of USTB-05-C was obtained (Figure 4). Asp184, His186 and His208 coordinated with Zn^2+^; Glu56 and Zn^2+^ cooperatively activated a water molecule and facilitated a nucleophilic attack on the hydroxyl oxygen atom of the Adda–Arg peptide bond; His150 formed a hydrogen bond with an oxygen atom to stabilize the transition states.

Furthermore, the above theoretical predictions were verified using the biodegradation kinetics. Not all strains were able to degrade the MCs and NODs [23]. Therefore, it was impossible that USTB-05-C had a stronger ability to degrade the NODs. However, MlrA of *Sphingopyxis* sp. USTB-05 cleaved the same Adda–Arg peptide bond in their cyclic peptide structure, and the rate for biodegrading NODs was obviously lower than that of MC-LR biodegradation [42]. Enzyme activity experiments showed that the linear NOD was completely degraded within 6 h by using purified MlrC.

Heterologous expressing and genetically engineered strains might be more efficient ways to degrade microcystins [9,43,44]. On the basis of these results, five mutation sites of MlrC were chosen, including Glu56, His150, Asp184, His186 and His208, for heterologous expression and purification. The five-point mutations in the zinc metalloprotease domain resulted in an almost complete loss of protease activity. The five-point mutations, Glu56, His150, Asp184, His186 and His208, were important activity sites for USTB-05-C to degrade MCs and NODs.

In this study, we successfully expressed and purified microcystinase MlrC originated from *Sphingopyxis* sp. USTB-05. Through combined computational and experimental approaches, a better understanding of the mechanism of linearized hepatotoxin biodegradation by MlrC was provided.

## 4. Conclusions

In this study, the recombinant USTB-05-C was obtained successfully with heterologous expression and purification. Its concentration reached up to 215 mg L^−1^ and was found to have a high activity in catalyzing hepatotoxins. The 3D structure of USTB-05-C was obtained with homology modeling. MlrC was deemed to be a metalloprotease with Zn^2+^. Bioinformatics analysis indicated that Zn^2+^ played an important role in stabilizing the MlrC structure and catalytic process. Glu56, His150, Asp184, His186 and His208 were the critical residues responsible for MlrC activity through site-directed mutagenesis.

## 5. Materials and Methods

### 5.1. Strain and Mutagenesis

*Sphingopyxis* sp. USTB-05 used in this study was isolated and identified by us previously [45]. Trifluoroacetic acids and methanol were purchased from Dikma Technology Inc. (Lake Forest, CA, USA) and used for HPLC analysis. *E. coli* TOP10 and *E. coli* BL21 (DE3) were purchased from Sangon Biotech Co., Ltd. (Shanghai, China). The recombinant and host strains were cultured in Luria–Bertani medium (tryptone 10 g, NaCl 10 g, yeast extract 5 g in 1 L H_2_O) in a shaker at 200 revolutions per minute (rpm) at 30 °C. The vector pET30a (+), restriction enzymes Sac I and Not I, plasmid mini-prep kit, polymerase chain reaction (PCR) kit and Ni-NTA Sefinose (TM) Resin Kit were also obtained from Sangon Biotech Co., Ltd. (Shanghai, China). Imidazole was purchased from Macklin Biochemical Co., Ltd. (Shanghai, China). NOD was purchased from Absin Bioscience Inc. (Shanghai, China) and stored at −20 °C. The purity of all other chemicals was no less than 99.7%.

### 5.2. Homology Modeling

MlrC (accession no.: AGH62481.1) originated from *Sphingopyxis* sp. USTB-05 was obtained from NCBI (https://www.ncbi.nlm.nih.gov/ (21 July 2022)) for bioinformatics analysis. MlrC consisted of 528 amino acids. The full-length sequences of MlrC with FASTA format were submitted to the Phyre^2^ engine for predicting the 3D structure. Other popular web servers for protein homology modeling, such as SWISS-MODEL and Geno3D, were examined for comparison as well. Only the results of Phyre^2^ were shown in the present report.

### 5.3. Cloning and Expression of Mutant Recombinants

Site-directed mutagenesis for the construction of six mutants, including MlrCE56A, MlrCH150A, MlrCD184A, MlrCH186A and MlrCH208A, was performed with overlap extension PCR. For detailed protocols concerning the oligonucleotide primers, see Sievers et al. [46]. The PCR products (MlrC mutants with full-length DNA) were digested by Sac I and Not I and ligated with the pET30a (+) vector that was digested by the same restriction enzymes as well. The recombinant plasmids were transformed into *E. coli* TOP10, and positive clones were screened out and confirmed with gene sequencing by Sangon Biotech Co., Ltd., (Shanghai, China). The confirmed plasmids were extracted from *E. coli* TOP10, followed by a transformation into an expression host of *E. coli* BL21 (DE3).

E. coli BL21 (DE3) cells were transferred with pET30a (+)-mlrC and plated on LB agar containing 50 μg ml^−1^ kanamycin. A single colony was cultured into liquid LB medium with 50 µg ml^−1^ kanamycin and shaken at a constant condition of 37 °C, 200 rpm. Isopropyl-β-D-thiogalactopyranoside (IPTG), with a final concentration of 0.2 mM, was added to induce protein expression when the optical density at 600 nm was approximately 0.6. Moreover, the bacterium was constantly incubated at 30 °C, 200 rpm for 3 h. One liter of recombinant cell culture was harvested using centrifugation (12,000× *g*, 4 °C, 25 min), washed thrice with PBS (pH 7.3) and resuspended in 20 mL PBS to a final concentration of 20–30 g dry cell weight per liter. The cells were disrupted with sonication (400 W, 20 min) in an ice bath and centrifuged (10,000× *g*, 4 °C, 30 min).

### 5.4. Purification of USTB-05-C

Total soluble protein concentration was used to evaluate the active MlrC expression level as shown above. Despite this, it was essential to obtain MlrC expression amount within the total soluble protein. The supernatant harvested in the last step was mixed with the column containing Ni-NTA sefinose resin (TaKaRa, Otsu, Japan). The recombinant enzyme MlrC bound to the column. The columns were washed with 5–10 column volumes of PBS (20 mM imidazole, pH 8.0) until the absorbance of the flow-through fraction at 280 nm approached the baseline. Then, the bound proteins were eluted from the columns with PBS (125 mM imidazole, pH 8.0). The protein profile was analyzed using sodium dodecyl sulfate-polyacrylamide gel electrophoresis (SDS-PAGE) on polyacrylamide gel. Moreover, the concentration of the purified protein was determined at 280 nm.

### 5.5. Determination of Enzyme Activity

To determinate the activity of the recombinant protein MlrC, a linear NOD was obtained by incubating a NOD with purified MlrA at 30 °C at the shaking rate of 200 rpm for 24 h [42]. Then, the linearized NOD was added to the PBS (PH 7.3) solution containing 1 mg L^−1^ of recombinant protein USTB-05-C and incubated at 30 °C, 200 rpm. The enzymatic reaction was stopped by adding 1% (*v*/*v*) HCl at 0 h, 2 h, 4 h, 6 h and 8 h, respectively. The active determination of the USTB-05-C mutants was in accordance with the steps for recombinant USTB-05-C mentioned above. Whole samples were centrifuged (12,000× *g*, 15 min, 4 °C) to monitor the linearized NOD concentrations with high-performance liquid chromatography (HPLC). The treated samples were analyzed with high-performance liquid chromatography (Shimadzu LC-20AT, Shimadzu Co., Ltd., Kyoto, Japan) with an ultraviolet diode array detector at 238 nm and a Purospher STAR RP-18 column (4.6 mm × 250 mm) (Merck KGaA, Darmstadt, Germany). The mobile phase was 40% (*v*/*v*) acetonitrile water solution containing 0.05% (*v*/*v*) of trifluoroacetic acid. The flow rate was 1.0 mL min^−1^ and the injection amount was 20 μL.

## Data Availability

Not applicable.
